# The Spasmolytic Effect of Astragalus Sarcocolla on the Intestinal Smooth Muscles of Rabbit In Vitro: Potassium Channel Opening

**DOI:** 10.7759/cureus.9066

**Published:** 2020-07-08

**Authors:** Waqar Ahmed Siddiqui, Muhammad Usama Mazhar, Javaria Arshad Malik, Aisha Talat, Sehrish Zaffar, Haroon Rashid, Irsa R Chattha

**Affiliations:** 1 Pharmacology, Combined Military Hospital (CMH) Lahore Medical College, National University of Medical Sciences (NUMS), Lahore, PAK; 2 Community Medicine, Combined Military Hospital (CMH) Lahore Medical College, National University of Medical Sciences (NUMS), Lahore, PAK

**Keywords:** astragalus sarcocolla, spasmolytic, potassium channel opener

## Abstract

Introduction

Astragalus species have been widely used in Chinese herbal medicine to treat gastrointestinal and inflammatory disorders. This study was conducted to evaluate the efficacy of *Astragalus sarcocolla* (ASE) and to rationalize its medicinal use as an antispasmodic drug for the treatment of spasmodic gastrointestinal and inflammatory disorders associated with increased intestinal motility.

Methods

The ethanolic extract of ASE was studied to examine its antispasmodic effect on the isolated rabbit ileum preparations, and the contractions were recorded on PowerLab (ADInstruments, Sydney, Australia).

Results

ASE was able to inhibit spontaneous ileum contractions. It also completely inhibited K^+^ (25 mM)-induced contractions but was unable to inhibit high K^+^ (80 mM)-induced sustained contractions. Pretreatment of the tissue with glibenclamide, a potassium channel blocker, caused a rightward shift of the dose-response curve when stimulated with K^+^ (25 mM) in the presence of an increasing concentration of the extract. Verapamil at very low doses inhibited both the 25 mM and 80 mM K^+^-induced contractions.

Conclusion

The results of our study demonstrated the spasmolytic activity of ASE with the potential mechanism of activation of K^+^ATP, which provides a strong basis for its medicinal use in motility and inflammatory disorders of the intestine.

## Introduction

Functional gastrointestinal disorders such as irritable bowel syndrome (IBS) are common disorders affecting scores of people worldwide. They are characterized by abdominal discomfort and pain, which are usually felt after food intake, and associated with an increase in the frequency of stools and alternating constipation. IBS is a chronic disorder with a relapsing and remitting natural history [1]. Studies have shown that IBS affects 10-20% of the adult population globally and also accounts for up to 25% of the outpatient workload of a gastroenterologist. It is obvious that the financial impact of this disorder on society is high due to medical costs, absenteeism from work, and impaired health-related quality of life [2]. The interest in the practical use of herbal substances and preparations for medicinal purposes has been growing steadily worldwide.

Astragalus species are extensively distributed throughout the temperate and arid regions. The genus is currently estimated to have 2,000-3,000 species [3-5]. More than 200 constituents have been obtained so far from the Astragalus genus. Though studies have been conducted on different species, the chemical composition of Astragalus genus extract has appeared highly uniform, comprising of saponins, flavonoids, and polysaccharides [6]. *Astragalus sarcocolla* Dymock belongs to the Fabaceae family [7]. Sarcocolla in Greek means “flesh glue”. It is a perennial herb that is unbranched and six feet long. Its resin, which is yellowish-brown in color, is known for its folk uses [8]. It is native to Iran and Turkistan but also found in India, Iraq, Kashmir, Kurdistan, Pakistan, and Western Himalayas [9]. *Astragalus sarcocolla *(ASE) has been used to treat putrescent wounds, eye diseases, paresis, weakness of sexual organs (aphrodisiacs), and epilepsy. Women have been using it to improve their skin. It is also used as an aperient and a fattening agent. Its antirheumatic, anthelmintic, and emollient effects are also well-known. Species of Astragalus have been widely studied and their roles in curing many medical ailments have been well-documented, especially relating to gastrointestinal disorders [8-12].

## Materials and methods

The ASE plant resin was obtained from the local herbarium market in Lahore, Pakistan in October 2019. The resin was ground to powder form using an electric grinder (Philips, Eindhoven, Netherlands) to facilitate the extraction steps. The powdered sample was stored in dry and clean conditions. The plant sample was identified by Dr. Hassan Mushtaq, a plant taxonomist from the department of Pharmacognosy, Punjab University, and was marked as voucher specimen no. 421 for further reference. The extraction was carried out by the cold extraction (maceration) method [13,14].

After that, 1 kg of powdered resin was immersed in 5 L of absolute ethanol for 72 hours at room temperature with occasional manual stirring. After 72 hours, the macerated plant material was filtered through a muslin cloth to separate vegetative debris. The obtained filtrate still contained some coarse particles of vegetative debris. The liquid obtained was further vacuum-filtered through a Büchner funnel using Whatman® filter paper No. 1 (Sigma-Aldrich, St. Louis, MO). The filtrate was evaporated under reduced pressure using rotary evaporator N-1000 (Eyela, Tokyo, Japan) at a temperature of 40 °C for one hour and then oven-dried until it turned into paste [15]. The dried extract was collected and stored in an airtight container in a refrigerator (2-4 °C) until use [16]. Phytochemical analysis of the ethanolic extract of ASE had been tested for the presence of alkaloids, flavonoids, sterols, terpenoids, saponins, and tannins [17].

We acquired the chemicals acetylcholine perchloride, magnesium chloride (MgCl_2_), potassium chloride (KCl), calcium chloride (CaCl_2_), and aluminum hydroxide (AlOH_3_) from Sigma-Aldrich Chemicals Company. Whereas glucose (C_6_H_12_O_6_), sodium bicarbonate (NaHCO_3_), sodium dihydrogen phosphate (NaH_2_PO_4_), magnesium sulfate (MgSO_4_), potassium dihydrogen phosphate (KH_2_PO_4_), and sodium chloride (NaCl) were purchased from E. Merck KG (Darmstadt, Germany). Verapamil hydrochloride was procured from Searle Pharmaceuticals (Karachi, Pakistan) and glibenclamide from Sanofi Aventis (Paris, France). All the chemicals used for this study were of analytical grade and dissolved in distilled water for the experiments.

Adult rabbits (either sex) of local breed weighing 1-1.5 kg were used in this study. Animals were housed at the animal house of Combined Military Hospital (CMH) Lahore Medical College, Lahore, Pakistan. The temperature range was set at 23-25 °C, humidity at 60% ± 4%, at 12/12 hours of light-dark cycles. Tap water and diet were provided ad libitum. The experiments performed were approved by the Ethical Committee of the CMH Lahore Medical College.

In order to evaluate the antispasmodic effect of ASE on isolated rabbit’s ileum, the rabbits of either sex were euthanized by using the method of cervical dislocation. Rabbits were dissected and ileum was carefully removed from the mesentery. The composition of Tyrode’s solution in mM was as follows: 5.55 glucose, 11.90 NaHCO_3_, 1.05 MgCI_2_, 136.9 NaCI, 1.8 CaCl_2_, 2.68 KCI, and 0.42 NaH_2_PO_4_ (pH 7.4); it was freshly made and the 2-cm long segments of ileum were immersed in tissue bath (Radnoti 159920-X1/C; Radnoti Llc, Covina, CA) containing 25 ml of Tyrode’s solution that was continuously aerated with carbogen (5% CO_2_ and 95% O_2_). After hanging the tissue, 1 g of resting tension was applied as preload. The tissue was allowed to equilibrate for 30 minutes before any drug was added to the tissue bath [18]. To record the intestinal contractions, an isotonic transducer MLT0015 (Panlab, Harvard Apparatus, Holliston, MA) was attached to the PowerLab (Model: PL26T04) data acquisition system (ADInstruments, Sydney, Australia). All the recordings were recorded on LabChart software version 8 (ADInstruments).

In order to stimulate and then stabilize the intestinal contractions, acetylcholine at a submaximal concentration of 0.3 μM was used at three-minute intervals until the recorded responses became stable. The spontaneous contractions were then recorded and the spasmolytic effect of drugs was analyzed without directly applying any agonist or antagonist drug. ASE was then added in a cumulative manner with the final concentration of 0.04-8 mg/ml into the tissue bath containing Tyrode’s solution to record its relaxant effect on the ileum [19].

For rationalizing the mechanism of ASE’s spasmolytic effect, the ileum was depolarized first with a solution containing potassium as KCl with final molar concentrations of low K^+^ (25 mM) and then with high K^+^ (80 mM) that produced sustained contractions. ASE was then added in a cumulative manner into both low K^+^ and high K^+^ contracted ileum and concentration-dependent inhibitory responses were recorded. After that, the same procedure was repeated in the presence of glibenclamide and verapamil. The relaxation of isolated tissue preparations was expressed as a percentage of the control response mediated by adding low and high K^+^ concentrations [20].

All the data were expressed in mean ± standard error of the mean (SEM, n=5) and the median effective concentrations (EC50 values) with 95% confidence intervals (CI). The concentration-response curves were analyzed by non-linear regression using GraphPad Prism version 5 (GraphPad Software, San Diego, CA).

## Results

**Figure 1:**
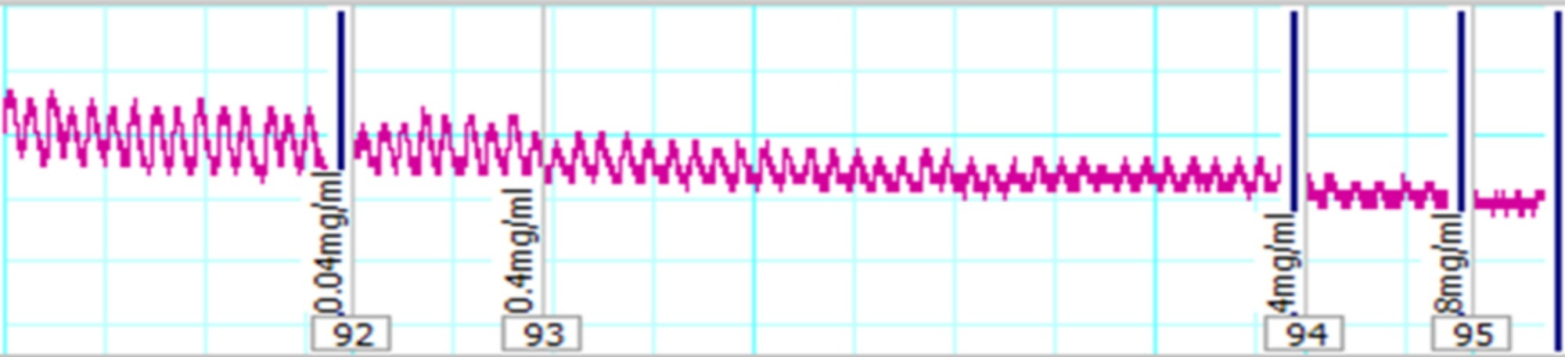
Tracing showing the relaxant effects of the crude extract of ASE (0.04-8 mg/ml) on spontaneous contractions of isolated rabbit ileum preparations ASE: *Astragalus sarcocolla*

ASE was investigated for its possible spasmolytic action on isolated rabbit ileum, and it was able to inhibit the spontaneous contractions as well as K^+^-induced sustained contractions in a dose-dependent manner. The spasmolytic effect on the spontaneous contractions was dose-dependent with an EC50 value of 3.6 mg/mL (95% CI: 0.04-3.4, n=5) (Figure 1).

**Figure 2:**
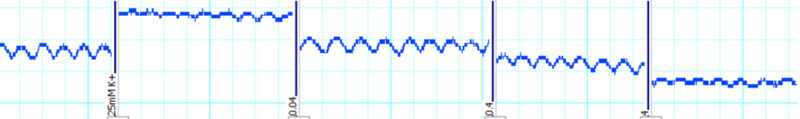
Tracing showing the relaxant effects of the crude extract of ASE (0.4-4 mg/ml) on low K+ (25 mM)-induced sustained contractions of isolated rabbit ileum preparations ASE: *Astragalus sarcocolla*

ASE was also evaluated for its spasmolytic effect on low K^+^ (25 mM)-induced contractions. ASE completely inhibited the low K^+^ (25 mM)-induced contractions with EC50 value of 0.06 mg/mL (95% CI: 0.04-0.16, n=5) (Figure 2).

**Figure 3:**
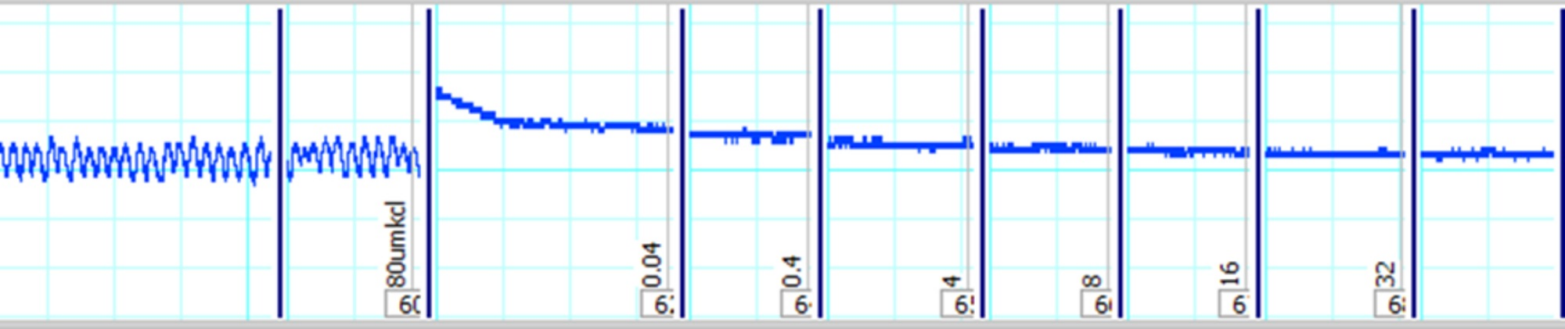
Tracing showing the relaxant effects of the crude extract of ASE (0.04-32 mg/ml) on high K+ (80 mM)-induced sustained contractions of isolated rabbit ileum preparations ASE: *Astragalus sarcocolla*

However, when ASE was tested against high K^+^ (80 mM)-induced contractions, only a mild inhibitory effect was observed even at higher doses (32 mg/mL) (Figure 3).

**Figure 4:**
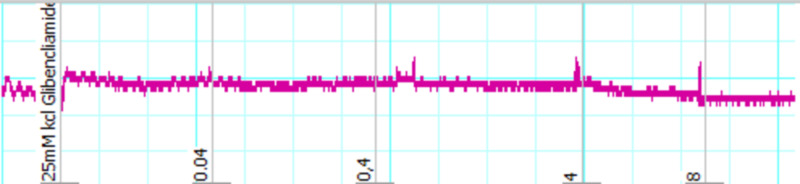
Tracing showing the blockage of relaxant effects of the crude extract of ASE (0.04-8 mg/ml) by glibenclamide on low K+ (25 mM)-induced sustained contractions of isolated rabbit ileum preparations ASE: *Astragalus sarcocolla*

**Figure 5:**
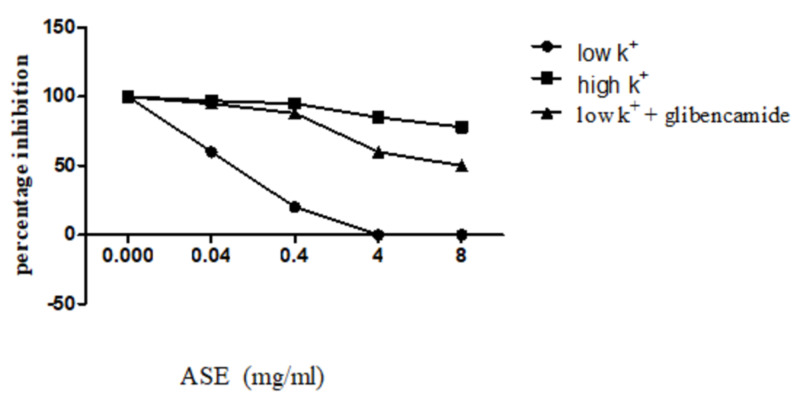
The spasmolytic effects of the crude extract of ASE on low (25 mM), high K+ (80 mM), and low K+ + glibenclamide-induced contractions in isolated rabbit ileum preparations Values shown represent mean ± SEM of 4-5 determinations ASE: *Astragalus sarcocolla*; SME: standard error of mean

For the confirmation of its K^+^ channel opening ability, glibenclamide was used and it prevented the relaxant effect of ASE, at the doses it inhibited the contractions in the absence of glibenclamide (Figures 4, 5).

**Figure 6:**
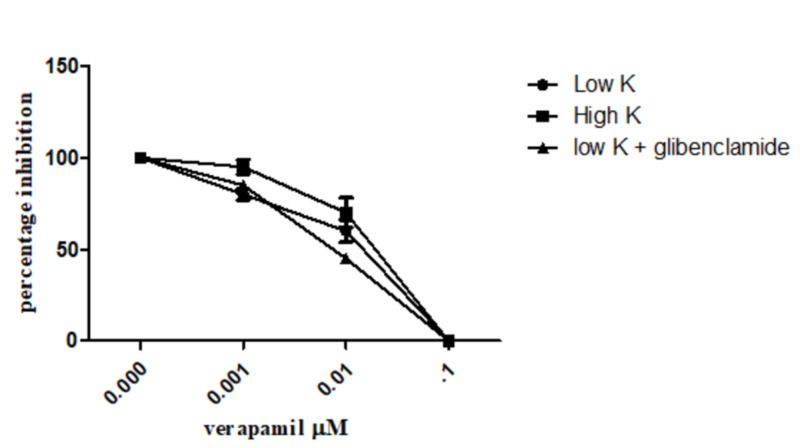
The spasmolytic effects of verapamil on low (25 mM), high K+ (80 mM), and low K+ + glibenclamide-induced contractions in isolated rabbit ileum preparations Values shown represent mean ± SEM of 4-5 determinations ASE: *Astragalus sarcocolla*; SME: standard error of mean

Verapamil, a standard Ca^2+^ channel blocker, inhibited low K^+^- and high K^+^-induced contractions at low (0.01-0.1 uM) concentrations (Figure 6).

## Discussion

Gastrointestinal diseases like IBS and inflammatory bowel disease are common health problems affecting millions of people worldwide. Both are debilitating conditions characterized by pain due to intestinal spasm and diarrhea. Drugs used to treat these conditions are usually taken for long durations, which often leads to side effects. In this study, ASE was tested for its antispasmodic effect, and it was found that it has serious antispasmodic potential. Most of the plants that are used as antispasmodics work mainly by two mechanisms: 1) by blocking the calcium channels in the intestines and causing relaxation and 2) by the opening of potassium channels and producing relaxant effect in the intestine [21].

ASE caused inhibition of spontaneous contractions of isolated ileum preparations. To assess the mechanism by which the spasmolytic effect was produced by ASE either by blockade of calcium channels or opening of potassium channels, the ASE extract was tested against high K^+^ (80 mM)-induced contractions. According to various studies, the possible mechanism of sustained contractions that are produced by high K^+^ is believed to be due to cell surface voltage-gated Ca^2+^ channels (VGCCs), which get activated by cell membrane depolarization and cause an increase in Ca^2+^ extracellularly. Ca^2+^ ions that enter through the cell through VGCCs may also trigger more Ca^2+^ release via ryanodine receptors (RyRs). Another way involves a scenario where depolarization is directly coupled to sarcoplasmic Ca^2+^ release channels. Depolarization-induced Ca^2+^ release (DICR) is known to be necessary for excitation-contraction coupling in the skeletal muscle and smooth muscles; the rise in Ca^2+^ leads to calmodulin-dependent activation of myosin light chain kinase (MLCK) and results in the phosphorylation of the 20-kDa light chain of myosin and eventually leads to the contraction of smooth muscles [22]. In our study, the negligible inhibitory effect of ASE against high K^+^-induced contractions (Figure 3) suggests that the spasmolytic effect is perhaps mediated through some other mechanism. ASE, when tested against low K^+^ (25 mM)-induced contractions, caused complete inhibition of the contractions produced by low K^+^ (Figure 2). Any substance that selectively relaxes the contractions induced by <25 mM K^+^ is considered a potassium channel opener [18].

When tested in the presence of glibenclamide, which is a specific blocker of the ATP-dependent K^+^ channels [23], the inhibitory effect of the ASE extract was found to be decreased (Figure 4). These results clearly and strongly indicate that the spasmolytic effect of the plant extract is mediated possibly through ATP-dependent K^+^ channel activation [19]. However, because of the non-availability of a standard potassium channel opener, ASE was not compared with it. Verapamil, a calcium channel blocker, showed inhibition of both low- and high K^+^-induced contractions at very low doses (Figure 6), which indicates ASE's ability to activate ATP-dependent K^+^ channels [24]. Activation of K^+^ channels in cell membranes results in an increase of K^+^ efflux and causes the shift of membrane potential towards hyperpolarization and directs towards the K^+^ equilibrium potential; as a result of hyperpolarization, the opening ability of ion channels that are involved in membrane depolarization is reduced along with a reduction in excitation, resulting in decreased intracellular free Ca^2+^ and smooth muscle relaxation [25]. The weak inhibitory effect of ASE on the sustained contractions induced by high K^+^ along with glibenclamide-sensitive relaxation of low K^+^-induced contractions strongly suggests the presence of K^+^ ATP channel-opening constituents in ASE.

The observed spasmolytic effect of isolated rabbit’s ileum is mediated through K^+^ channel activation. This may explain its use in spastic gut disorder, and further studies may prove its anti-inflammatory effects as well.

## Conclusions

This study concludes that ASE decreases in-vitro intestinal contractions. The observed spasmolytic effect of isolated rabbit’s ileum is mediated through K^+^ channel activation.

## References

[1] Halpin SJ, Ford AC (2012). Prevalence of symptoms meeting criteria for irritable bowel syndrome in inflammatory bowel disease: systematic review and meta-analysis. Am J Gastroenterol.

[2] Ghoshal UC, Abraham P, Bhatt C (2008). Epidemiological and clinical profile of irritable bowel syndrome in India: report of the Indian Society of Gastroenterology Task Force. Indian J Gastroenterol.

[3] Podlech D (2008). The genus Astragalus L. (Fabaceae) in Europe with exclusion of the former Soviet Union. Feddes Repert.

[4] Benchadi W, Haba H, Lavaud C, Harakat D, Benkhaled M (2013). Secondary metabolites of Astragalus cruciatus Link and their chemotaxonomic significance. Rec Nat Prod.

[5] Xu L, Podlech D (2010). Astragalus. Flora of China.

[6] Ibrahim LF, Marzouk MM, Hussein SR, Kawashty SA, Mahmoud K, Saleh NA (2013). Flavonoid constituents and biological screening of Astragalus bombycinus Boiss. Nat Prod Res.

[7] Nadkarni KM (1996). Indian Materia Medica With Ayurvedic, Unani-Tibbi, Siddha, Allopathic, Homeopathic, Naturopathic & Home Remedies. Nadkarni's Indian materia medica : with Ayurvedic, Unani-Tibbi, Siddha.

[8] Lev E, Amar Z (2008). Practical Materia Medica of the Medieval Eastern Mediterranean According to the Cairo Genizah. Brill.

[9] Drug Information System. Astragalus sarcocolla Dymock 2014 [cited 2017 13 April ]. Available from: http://druginfosys.com/herbal/Herb.aspx?Code=622&Name=Astragalus%20sarcocolla%20Dymock&type=1 (2017). Drug Information System: Astragalus sarcocolla Dymock. http://druginfosys.com/herbal/Herb.aspx?Code=622&Name=Astragalus%20sarcocolla%20Dymock&type=1.

[10] Watt G (1889). A Dictionary of the Economic Products of India. https://books.google.co.in/books?id=89ku-rP4aS8C&source=gbs_book_other_versions.

[11] Academia Nacional de Ciencias (1970). Boletín: Academia Nacional de Ciencias. Boletín de la Academia Nacional de Ciencias.

[12] Khare CP (2008). Indian Medicinal Plants: An Illustrated Dictionary. Indian Medicinal Plants: An Illustrated Dictionary: Springer New York.

[13] Williamson EM, Okpako DT, Evans FJ (1996). Selection, Preparation and Pharmacological Evaluation of Plant Material. https://www.wiley.com/en-us/Selection%2C+Preparation+and+Pharmacological+Evaluation+of+Plant+Material%2C+Volume+1-p-9780471942177#:~:text=1%3A%20Selection%2C%20Preparation%2C%20and,investigation%20and%20presentation%20of%20results..

[14] Yuet Ping K, Darah I, Yusuf UK, Yeng C, Sasidharan S (2012). Genotoxicity of Euphorbia hirta: an Allium cepa assay. Molecules.

[15] Patel VR, Patel PR, Kajal SS (2010). Antioxidant activity of some selected medicinal plants in western region of India. Adv Biol Res.

[16] Bukhari IA, Khan RA, Gilani AH, Ahmed S, Saeed SA (2010). Analgesic, anti-inflammatory and anti-platelet activities of the methanolic extract of Acacia modesta leaves. Inflammopharmacology.

[17] Bratkov VM, Shkondrov AM, Zdraveva PK, Krasteva IN (2016). Flavonoids from the genus Astragalus: phytochemistry and biological activity. Pharmacogn Rev.

[18] Gilani AH, Mehmood MH, Janbaz KH, Khan AU, Saeed SA (2008). Ethnopharmacological studies on antispasmodic and antiplatelet activities of Ficus carica. J Ethnopharmacol.

[19] Imtiaz SM, Aleem A, Saqib F, Ormenisan AN, Neculau AE, Anastasiu CV (2019). The potential involvement of an ATP-dependent potassium channel-opening mechanism in the smooth muscle relaxant properties of Tamarix dioica Roxb. Biomolecules.

[20] Mehmood MH, Hassan W (2015). Antidiarrheal and antispasmodic activities of Adiantum capillusveneris are predominantly mediated through ATP-dependent K+ channels activation. Bangladesh J Pharmacol.

[21] Uchida Y, Maezawa Y, Maezawa Y, Uchida Y, Nakamura F (2011). Role of calcium-activated potassium channels in the genesis of 3,4-diaminopyridine-induced periodic contractions in isolated canine coronary artery smooth muscles. J Pharmacol Exp Ther.

[22] Kirschstein T, Rehberg M, Bajorat R, Tokay T, Porath K, Köhling R (2009). High K+-induced contraction requires depolarization-induced Ca2+ release from internal stores in rat gut smooth muscle. Acta Pharmacol Sin.

[23] Luzi L, Pozza G (1997). Glibenclamide: an old drug with a novel mechanism of action?. Acta Diabetol.

[24] Kimura S, Bassett AL, Xi H, Myerburg RJ (1992). Verapamil diminishes action potential changes during metabolic inhibition by blocking ATP-regulated potassium currents. Circ Res.

[25] Cheng CJ, Kuo E, Huang CL (2013). Extracellular potassium homeostasis: insights from hypokalemic periodic paralysis. Semin Nephrol.

